# Visualising the Future of Orthopaedic Surgery: A Novel Application of Wireless Smart Glasses to Visualise Intraoperative Imaging

**DOI:** 10.7759/cureus.22004

**Published:** 2022-02-08

**Authors:** Se Ri Park, Jae Yong Park, Rafia Ghani, Joon Ha, Thomas Hester

**Affiliations:** 1 Medicine and Surgery, Imperial College London, London, GBR; 2 Trauma and Orthopaedic Surgery, Guy’s and St. Thomas’ NHS Foundation Trust, London, GBR; 3 Trauma and Orthopaedic Surgery, Guy's and St. Thomas' NHS Foundation Trust, London, GBR; 4 Trauma and Orthopaedic Surgery, King's College Hospital NHS Foundation Trust, London, GBR

**Keywords:** surgical ergonomics, assistive technology, heads-up display, smart glasses, intraoperative imaging

## Abstract

Smart glasses can provide a heads-up display of advanced imaging intraoperatively. In recent years, growing attention has been drawn to the use of smart glasses as an assistive technology to improve both efficiency and ergonomics in a surgical setting. Previous studies have reported improved surgical accuracy, efficiency, and ergonomics with its usage, but its effectiveness as a form of intraoperative heads-up display remains elusive in the context of orthopaedics. This study provides a novel account of a wireless set-up of the Moverio BT-35E Smart Glasses (Suwa, Japan: Epson Inc.), incorporated in a complex orthopaedic procedure.

Hind-foot nailing was performed on a patient with a complex open ankle fracture and multiple co-morbidities. Smart glasses were worn by the primary surgeon throughout the procedure to provide heads-up visualisation of the intraoperative fluoroscopy.

In our surgical case, the surgeon experienced improved ergonomics and reduced disruption to focus with the use of smart glasses. The wireless set-up provided excellent signal transmission throughout the duration of the procedure.

The wireless set-up of smart glasses is a potential solution for common occupational risks imposed on orthopaedic surgeons. Smart glasses minimise musculoskeletal strain from switching of vision from monitor to patient, whilst the wireless set-up allows for efficient use of space in an operating theatre and may potentially limit radiation exposure. Lastly, ergonomic benefits may increase the efficiency of movement for the surgeon, decreasing operative duration, and in turn minimising the risk of surgical complications for patients.

## Introduction

The use of intraoperative imaging is common in orthopaedic surgery. However, since the first account of its usage in the 1950s, there has been limited innovation. Only recently, with the increasing surgeon and patient demands for improvement, has the advancement of technology begun to parallel our expectations for innovation. We report a novel account of the first-of-its-kind wireless set-up of intraoperative imaging viewed through a heads-up display, which enables improved ergonomic experience for surgeons. We also provide insight into its application to a complex orthopaedic procedure and discuss challenges that may require further investigation. 

Orthopaedic surgeries are often complex procedures that require a composite of intricacy and physical workload. The unique characteristics of non-ergonomic positions that orthopaedic surgeons have to adapt to inevitably put them at a higher risk of occupational injuries such as musculoskeletal disorders [1,2]. Furthermore, the repetitive diversion of attention from the operative field to the monitor screen often contributes to reduced efficiency of procedures and may aggravate susceptibility to surgical errors. Recently, the application of wearable technology has sparked great interest within the medical field as a potential solution to these remaining challenges in a surgical setting. Notably, smart glasses provide a heads-up display of intraoperative images or videos within the surgeon’s field of view and encase potential benefits of its intraoperative usage. Although its usage has been tested in various surgical specialties such as vascular and neurosurgery with encouraging results, the effectiveness of its application in orthopaedic procedures is unclear [3-7]. Additionally, the reliability of a wireless high-definition multimedia interface (HDMI) signal transmission requires further validation. Using the Moverio BT-35E Smart Glasses (Suwa, Japan: Epson Inc.), this case provides the framework for evaluating the potential value of smart glasses as an assistive device during orthopaedic procedures.

## Technical report

**Figure 1:**
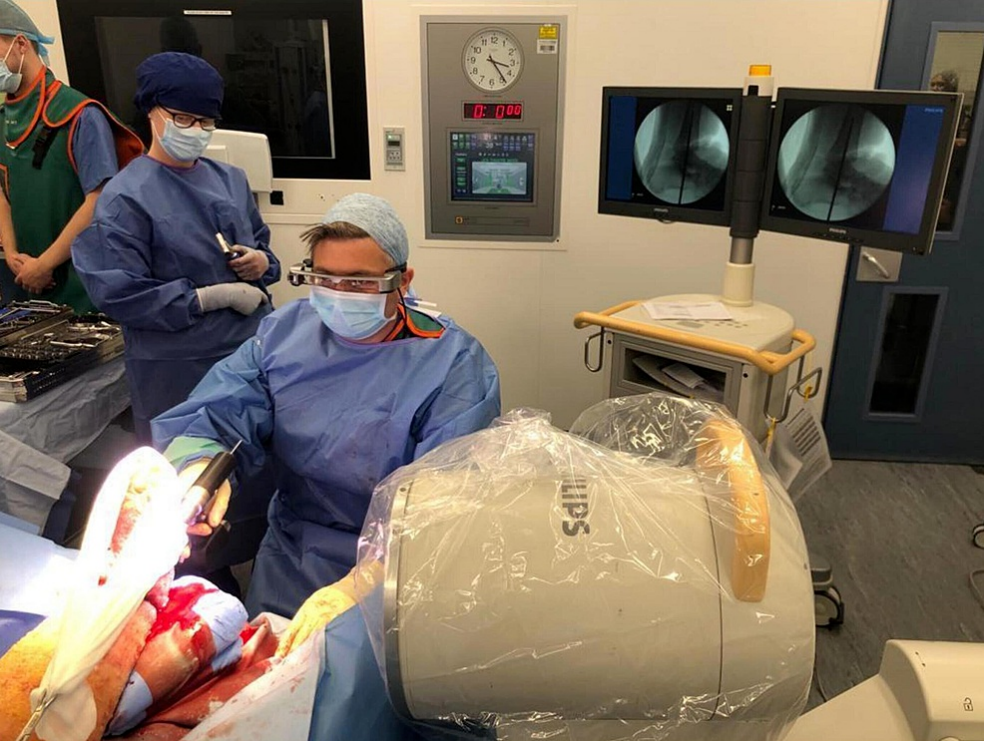
Surgical setting depicting the primary surgeon equipped with smart glasses and a receiver

We present a case of an elderly lady with an open ankle fracture in the presence of chronic lymphoedema, recurrent cellulitis, type 2 diabetes mellitus, and poor mobility. Taking into account her co-morbidities, the decision was made to shorten through the tibia to allow for primary soft tissue closure and hindfoot nail for early weight-bearing. After standard positioning on a radiolucent extension, the fracture and soft tissues were debrided by orthopaedic and plastic surgeons. The smart glasses and receiver were worn by the primary surgeon (Figure 1). 

Due to the nature of the injury and the availability of space in the operating theatre, monitors were placed against the back wall, and the C-arm (BV Endura; Eindhoven, the Netherlands: Philips) was brought in from the lateral side. This allowed for circumferential working around the limb, especially for fracture reduction, insertion of locking bolts and importantly, the nail entry point. With the use of smart glasses, positioning of the C-arm monitors became less critical as the active screen could be displayed in real-time via the lens. The glasses were ergonomically positioned so that they did not interfere with the surgical field of view and allowed the user to easily look between both the fluoroscopic image and the surgical field. This is critical during nail entry point insertion as this step has the greatest importance on final ankle positioning. Once the entry point was satisfactory, the canal was reamed sequentially and the nail placed in standard fashion.

**Figure 2:**
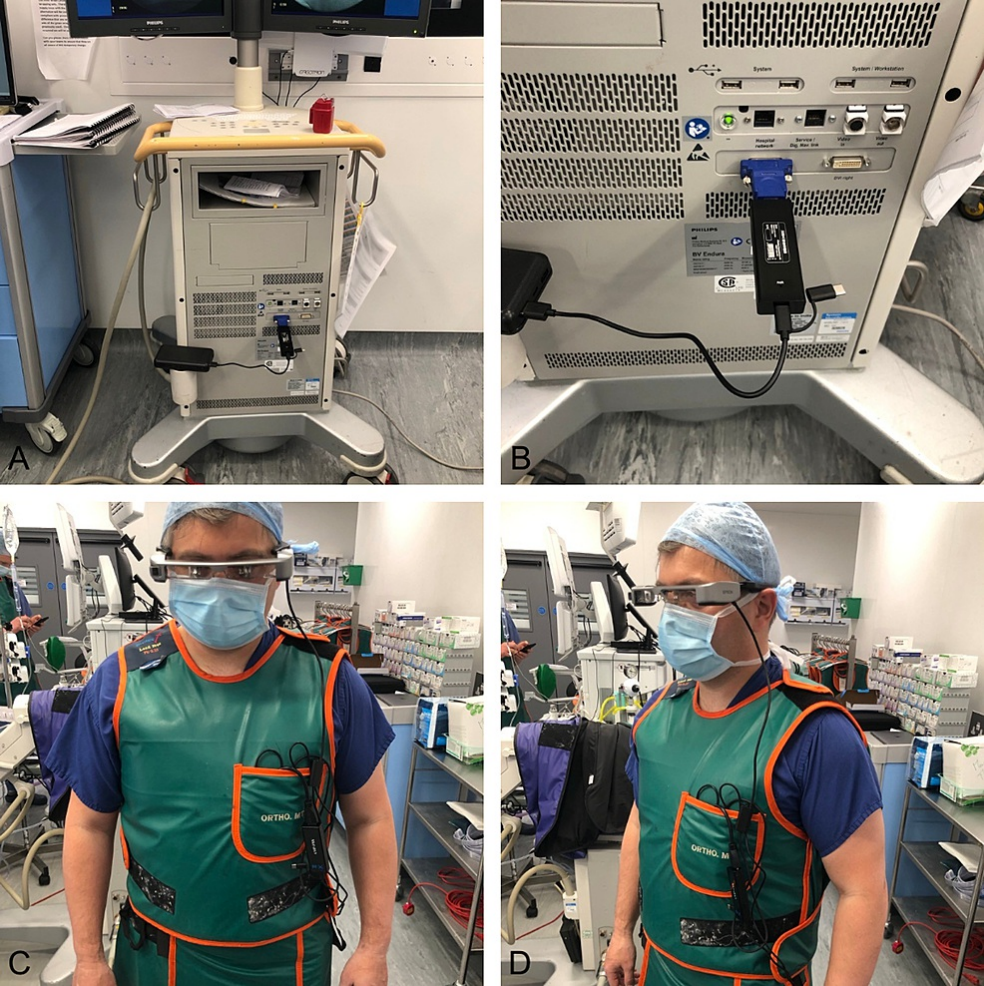
Wireless set-up of smart glasses in a surgical setting (A) Wireless set-up of the HDMI transmitter. (B) Close-up view of the wireless set-up of the HDMI transmitter depicting a Philips Endura image intensifier connected to a DVI-to-HDMI converter, which was then connected to a Measy wireless transmitter and a portable power bank. (C and D) Anterior and lateral view of the equipped smart glasses and wireless receiver connected to a battery pack.

The technical set-up included the Moverio BT-35E Smart Glasses, connected to a Measy Wireless HDMI Receiver (Shenzhen, China: Measy Technology Co., Ltd) and a portable 26800 mAh power bank. Connected to the Philips Endura image intensifier (Eindhoven, The Netherlands), via the digital visual interface (DVI) output was a DVI-to-HDMI converter, which was in turn connected to the Measy Wireless HDMI Transmitter and a portable 26800 mAh power bank (Figure 2).

## Discussion

This study provides a vital insight into how effective the incorporation of smart glasses can be in orthopaedic surgery. We report improvements in both ergonomics and surgical visualisation with the use of smart glasses, which corroborates findings from previous literature. To our knowledge, this is the first study to adopt a wireless set-up in an orthopaedic procedure, and we report excellent wireless connection of the smart glasses.

A pilot study conducted by Chimenti and Mitten reported significant improvement in the time efficiency of percutaneous pinning of hand fractures. With the use of smart glasses, the average time to pin phalangeal fractures was significantly reduced from 127.8 seconds to 86.8 seconds and required significantly fewer fluoroscopic images compared to a standard set-up of fluoroscopic visualisation [8]. Tsubosaka et al. also showed improvement in the accuracy of wire insertion during fracture surgeries with the utilisation of smart glasses [9]. When smart glasses were utilised to insert a K-wire into an artificial bone, a significant reduction in the mean error angle (4.8° vs 3.2°) was reported, providing insight into the contribution of its navigational features towards surgical precision [9].

Meanwhile, other studies evaluating the use of smart glasses compared to the standard surgical set-up have provided evidence for the safe usage of wearable smart glasses in achieving comparable patient outcomes to current gold standard procedural settings [10,11]. Unanimously, these studies have emphasised the reduced disruption to the operative field and maintenance of a focused workflow with the use of smart glasses, reiterating the key advantages that have been highlighted throughout our study.

In contrast, there is a remaining scarcity of literature demonstrating a wireless set-up of wearable smart glasses. However, this study demonstrates a novel proof of concept attempt of a wireless set-up of smart glasses. In many previous studies, long cable tethers were the main form of connection between the wearable smart glasses and the heads-up display, but in operating theatres with a large surgical team and multiple monitors, the presence of transmitter cables may pose a trip hazard. Taking into account safety precautions and the importance of efficient use of space in an operating theatre, a wireless HDMI transmitter was used in an attempt to evaluate both the reliability and mobility of the wireless set-up. Good signal was achieved throughout the entirety of the procedure without technical disturbances, alluding to a successful wireless adaptation of smart glasses in operating theatres.

Effect on ergonomics

Our experience with the Moverio BT-35E Smart Glasses presents enthusiastic findings that corroborate previous studies to show improved ergonomic effects. The ability to make fine adjustments without moving or looking away from the surgical field was replicable, and with minimal movement, good patient outcomes were achieved. Through improving the surgical ergonomics of orthopaedic procedures, the aforementioned benefits of increased efficiency and reduced operative duration are likely to follow, reducing the risk of potential surgical complications that may worsen the prognosis of patients at an economic cost under USD 1000 [12]. 

Occupational hazards

Occupational injuries are common amongst orthopaedic surgeons. Given this, smart glasses may contribute more effective advantages in orthopaedics compared to other surgical specialties. Surveys report that 66.7% of orthopaedic surgeons have work-related musculoskeletal disorders. Furthermore, given that orthopaedic surgeons have a high reliance on projected X-ray imaging during procedures, the constant shifting of vision from patient to monitor may be contributory to aggravation of this dilemma [1,2].

Intraoperative radiation exposure is another key occupational hazard to orthopaedic surgeons [13,14]. Compared to workers unexposed to routine radiation, orthopaedic surgeons were found to be more than five times likely to develop cancer [15]. Intraoperative assessment of reduction and implant position during treatment of fractures often require mobile C-arms or other imaging modalities which emit ionising radiation. This ionising radiation attributes to the high radiation exposure, leading to a high risk of cancer development amongst orthopaedic surgeons [16]. However, with the use of smart glasses potentially improving the time efficiency of operations, these shorter operation times would enable surgeons to shorten their exposure to radiation. This could significantly reduce radiation risk to medical professionals. More specifically, the risk of developing cancer.

Hence from an orthopaedic point of view, the potential advantages of the use of smart glasses are evident. If proven to be effective, smart glasses encase the potential to improve both ergonomic posture and positioning during surgical procedures and reduce the risk of occupational hazards commonly imposed on orthopaedic surgeons. 

Limitations

However, several limitations were also identified. The tested smart glasses were only able to project a single screen image. Hence in procedures where a comparison between multiple images is required, there may be limited benefits to the surgical ergonomics. Additionally, although projected images were sharp and prominent on darker backgrounds, on blue drapes or the patient, visual acclimation was required for the delivery of a focused image. To overcome this, further technological developments allowing potential incorporation of a multi-view mode or automatic brightness control may build on the advantages of smart glasses specific to a surgical setting. 

## Conclusions

This novel report reflects on the effective visualisation of intraoperative images with the incorporation of smart glasses in orthopaedic operating rooms via a wireless set-up. We also demonstrate the benefits of improved ergonomics and surgical concentration with its usage. Our experiences reflect the potential for increased usage of smart glasses to contribute incremental changes that may lead to revolutionary changes in the field of orthopaedic surgery. Similar wearable technologies with augmented reality are still in their early stages of validation and larger comparative studies or case series expanding on diverse clinical cases and surgical experience are vital before smart glasses can form part of a standard surgical set-up.
